# Learning adaptive representations for entity recognition in the biomedical domain

**DOI:** 10.1186/s13326-021-00238-0

**Published:** 2021-05-17

**Authors:** Ivano Lauriola, Fabio Aiolli, Alberto Lavelli, Fabio Rinaldi

**Affiliations:** 1 Department of Mathematics, University of Padova, Via Trieste 63, Padova, 35121 Italy; 2 Fondazione Bruno Kessler, Via Sommarive 18, Trento, 38123 Italy; 3 Dalle Molle Institute for Artificial Intelligence Research (IDSIA), Via Cantonale 2C, Manno, 6928 Svizzera; 4 Department of Quantitative Biomedicine, University of Zurich, Andreasstrasse 15, Zürich, 8050 Svizzera; 5 SIB, Swiss Institute of Bioinformatics, Génopode, Quartier UNIL-Sorge, bâtiment, Lausanne, 1015 Svizzera

**Keywords:** Named entity recognition, Neural networks, Kernel methods, Ensemble

## Abstract

**Background:**

Named Entity Recognition is a common task in Natural Language Processing applications, whose purpose is to recognize named entities in textual documents. Several systems exist to solve this task in the biomedical domain, based on Natural Language Processing techniques and Machine Learning algorithms. A crucial step of these applications is the choice of the representation which describes data. Several representations have been proposed in the literature, some of which are based on a strong knowledge of the domain, and they consist of features manually defined by domain experts. Usually, these representations describe the problem well, but they require a lot of human effort and annotated data. On the other hand, general-purpose representations like word-embeddings do not require human domain knowledge, but they could be too general for a specific task.

**Results:**

This paper investigates methods to learn the best representation from data directly, by combining several knowledge-based representations and word embeddings. Two mechanisms have been considered to perform the combination, which are neural networks and Multiple Kernel Learning. To this end, we use a hybrid architecture for biomedical entity recognition which integrates dictionary look-up (also known as gazetteers) with machine learning techniques. Results on the CRAFT corpus clearly show the benefits of the proposed algorithm in terms of *F*_1_ score.

**Conclusions:**

Our experiments show that the principled combination of general, domain specific, word-, and character-level representations improves the performance of entity recognition. We also discussed the contribution of each representation in the final solution.

## Background

The constant growth of the biomedical literature requires increasingly complex methods to index, categorize and retrieve documents from large-scale online databases and repositories. The aim of Named Entity Recognition (NER) [1] is to recognize and extract relevant entities and concepts from text. The extraction of such entities could help large-scale searching algorithms to semantically index and retrieve relevant documents. NER can be performed either on general texts (e.g., newspaper articles), to recognize general concepts like person, organization, or location, or on technical documents to recognize domain-specific concepts. In the biomedical domain (e.g. biomedical scientific literature), entities and concepts can be names of proteins, cellular components, diseases, species and so on. Moreover, NER is a preliminary step for other more complex tasks, such as relation extraction, sentiment analysis, dialogue and knowledge-base maintenance.

Even if the recognition of entities could be trivial for a human, automatic algorithms face several issues on these tasks, due to the complexity of the human language, the presence of ambiguity, and the unstructured characteristic of texts and documents. The first difficulty of biomedical NER (BNER) concerns the ambiguity of the terms, which can refer to multiple concepts. The recognition of a term strongly depends on the context to which it belongs. A classical example is provided by the token *CAT*. This token could be relevant if the system is looking for species and/or common names of animals. Nevertheless, *CAT* is also the acronym for *Computed Aided Tomography* or for *Chloramphenicol Acetyl Transferase*. Hence the same term could be a relevant entity in case the system wants to recognize acronyms or medical procedures. Another issue in this task is that proteins and other biomedical entities can be written in different ways. E.g. the *“human immunodeficiency virus”* may be written explicitly or by using acronyms, such as “HIV-1”, “HIV 1” or “H.I.V 1”. Further sources of difficulties include abbreviations, errors and the occurrences of novel entities.

Natural Language Processing (NLP) techniques have been widely used in the literature to solve this task [1]. Classical approaches include the usage of domain-specific manually defined rules which are able to recognize entities in documents. These rules can be regular expressions of particular characteristics of the entities. Another simple approach is based on dictionary look-up, and it finds the occurrences of entities in a document from a precompiled dictionary or ontology, which contains all of the possible entities. However, there are some issues with these methods, such as the human effort to maintain the dictionary, and the difficulty of designing powerful and effective rules. Recently, Machine Learning algorithms have been applied on this task [2, 3] aiming to improve the performance of automatic BNER annotators. These systems mainly include the usage of neural networks [4, 5], Support Vector Machines (SVM) [6] and Conditional Random Fields (CRF) [7, 8].

On the one hand, these mechanisms reduce human effort in designing adequate and expressive rules showing good results with domain-specific features. On the other hand, these methods inject further problems, such as the need of annotated data to feed the algorithms, the computational cost, and the definition of the data representation which describes tokens and entities. The choice of the data representation is a hard task for biomedical NER (BNER) and Machine Learning applications in general. For instance, an entity can be represented as the set of characters that compose it, or as the set of documents in which it occurs.

It is well known [9] that different representations emphasize different aspects of the problem, and they provide different results. Hence, the selection of the representation is a key step for building a powerful predictor. A model-selection procedure is usually performed to choose the representation, where a set of predefined representations are evaluated on a validation (or development) set. The representation that achieves the highest score is used to train the model. However, this procedure is expensive when the number of possible representations is large, and the selection may be subject to prior bias, bounding the expressiveness of the learning system. Moreover, each representation has its own advantages. General-purpose word embeddings, such as the popular Word2Vec [10], can be easily pre-trained on large-scale corpora, and they do not require a lot of prior knowledge. Hand-crafted representations, instead, could better represent the problem by means of powerful prior knowledge, but they require a lot of human effort to extract relevant features. Since different representations express different, and virtually orthogonal, information, the cooperation between them could further improve the performance.

In this work, knowledge-based, deep, ad-hoc and general-purpose representations are combined together to improve the accuracy of a BNER system. The combination has been carried out by using two representation learning paradigms. The first is the Multiple Kernel Learning [11], whose purpose is to learn the representation as a principled combination of several base representations. The second consists of popular neural networks with specialized architectures.

As a proof-of-concept, the multi-representation methodologies have been integrated into a reference hybrid BNER system [3], showing the benefits of the combination of multiple representations applied to the CRAFT [12] corpus. In short, this hybrid system combines two different approaches. Firstly, a dictionary look-up is applied to the input documents to find candidate entities. Then, a representation is computed for each candidate, and a machine learning classifier is used to filter the set of candidates.

## Methods

This section introduces and describes the methods and algorithms used in this work, i.e. neural networks, kernel methods, the hybrid BNER architecture, the corpus, and the feature sets.

### Multiple kernel learning

Kernel Machines are a large family of Machine Learning algorithms widely used in the literature to solve classification, regression and clustering problems [13]. A kernelized algorithm, such as the popular SVM, comprises two elements. The first element is the learning algorithm whose solution is expressed by dot-products between training examples. The second consists of a symmetric positive semi-definite kernel function $k : \mathcal {X} \times \mathcal {X} \rightarrow \mathbb {R}$, which computes the dot-product in a Reproducing Kernel Hilbert Space (RKHS). This means that there is a function $\phi : \mathcal {X} \rightarrow \mathcal {K}$ which maps data from the input space $\mathcal {X}$ to the kernel space $\mathcal {K}$ such that *k*(***x***_*i*_,***x***_*j*_)=〈*ϕ*(***x***_*i*_),*ϕ*(***x***_*j*_)〉, where $\boldsymbol {x}_{i},\boldsymbol {x}_{j} \in \mathcal {X}$. The kernel implicitly defines data representation. Usually, expert users choose the kernel function exploiting their domain-specific knowledge, or via a validation procedure.

Recently the literature showed mechanisms to learn the kernel function directly from the training data. One of the most popular kernel learning paradigms is the Multiple Kernel Learning (MKL) [11], which learns the kernel as a principled combination of *P* base kernels. These base kernels correspond to different sources, or different notions of similarity between examples. Usually, linear non-negative combinations are used, with the form:

$$k_{\boldsymbol{\mu}}(\boldsymbol{x}_{i},\boldsymbol{x}_{j}) = \sum_{r=1}^{P}\mu_{r} k_{r}(\boldsymbol{x}_{i},\boldsymbol{x}_{j}),\quad \mu_{r} \ge 0 $$

where *k*_*r*_ is the *r*-th kernel function defined on the *r*-th representation *ϕ*_*r*_, and ***μ*** is the weights vector that the MKL learns, which defines the contribution of each base kernel to the final solution.

Several MKL algorithms exist in the literature, which differs each other for their combination mechanisms, objective function, regularization techniques and optimization procedure. Usually, these algorithms find the combination which maximizes a quality criterion of the resulting representation rather than an empirical loss, as is the case of neural networks. In this work, the EasyMKL [14] algorithm has been considered due to its empirical effectiveness and efficiency. In short, EasyMKL learns the linear non-negative combination of base kernels which maximizes the minimum distance between the positive and negative classes, i.e. the margin, that is:

$$\max_{\boldsymbol{\mu}}\ \min_{\boldsymbol{\gamma}}\ (1-\lambda) \boldsymbol{\gamma}^{\top} \boldsymbol{Y} \left(\sum_{r=1}^{P} \mu_{r} \boldsymbol{K}_{r}\right) \boldsymbol{Y} \boldsymbol{\gamma} + \lambda\|\boldsymbol{\gamma}\|_{2}^{2} $$

where ***Y*** is a diagonal matrix containing labels (*y*_*i*_∈{+1,−1}), ***K***_*r*_ is the *r*-th kernel matrix, and $\boldsymbol {\gamma } \in \{\gamma _{i} \ge 0, \sum _{i : y_{i}=1} \gamma _{i} = 1 \land \sum _{i : y_{i}=-1} \gamma _{i} = 1 \}$ is a probability distribution of positive and negative examples. *λ*∈[0,1] is a hyper-parameter of the algorithm which regularizes the combination. When *λ*=0, then the algorithm tries to maximize the margin without taking into account the regularization term, whereas when *λ*=1 the algorithm maximize the distance between the centroids of the positive and the negative classes. However, a relaxation of the problem is performed to make it tractable. See [14] to get more details concerning the optimization process.

The MKL framework has been widely used in the literature. Some examples of MKL applications in the biomedical domain are Metabolite identification [15], cancer sub-tipe discovery [16], and data-fusion in general [17].

In the remainder of this article, we use the acronym SVM to refer the Support Vector Machine trained on a single kernel, whereas MKL refers to the ensemble composed by EasyMKL to learn the kernels combination, and the SVM to solve the machine learning problem with the combined kernel. We used the implementation of SVM from the Scikit-learn [18] library. The implementation of EasyMKL comes from the MKLpy project, and it is freely available on its GitHub repository^1^.

### Neural networks

Neural networks are a popular class of learning algorithms inspired by the biological neural networks and astrocytes that constitute animal brains, and they have been widely used to solve a plethora of machine learning tasks. Some relevant examples of neural networks applications in the biomedical domains [19] are image segmentation [20], neuroimaging [21], and text classification [22].

From a computational point of view, neural networks rely on a stacked sequence of non-linear transformations which provides an increasingly complex representation of data [23]. Each transformation maps an input example in the next layer. The initial and the last layers are called input and output layers, whereas the other layers are named hidden. The layers, mappings, and neurons define the architecture of a neural network. In this work, we use fully-connected feed-forward neural networks (NN).

**Fig. 1:**
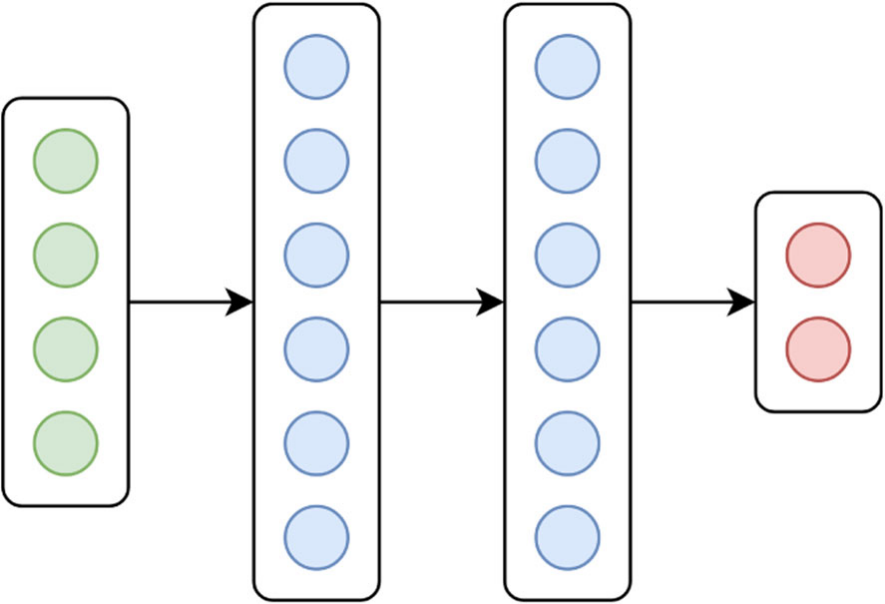
A depiction of a simple neural network. Arrows define the non-linear transformations from the input (green) to the output layer (red). Hidden layers are described in blue. Circles denote atomic features (or *neurons*)

The Fig. 1 depicts a general example of such architecture.

One of the main advantages of neural networks is their adaptability to different scenarios through the definition of a suitable architecture for a given problem. This aspect allows to define neural networks able to learn from different sources, mimicking the concepts of the MKL framework.

**Fig. 2:**
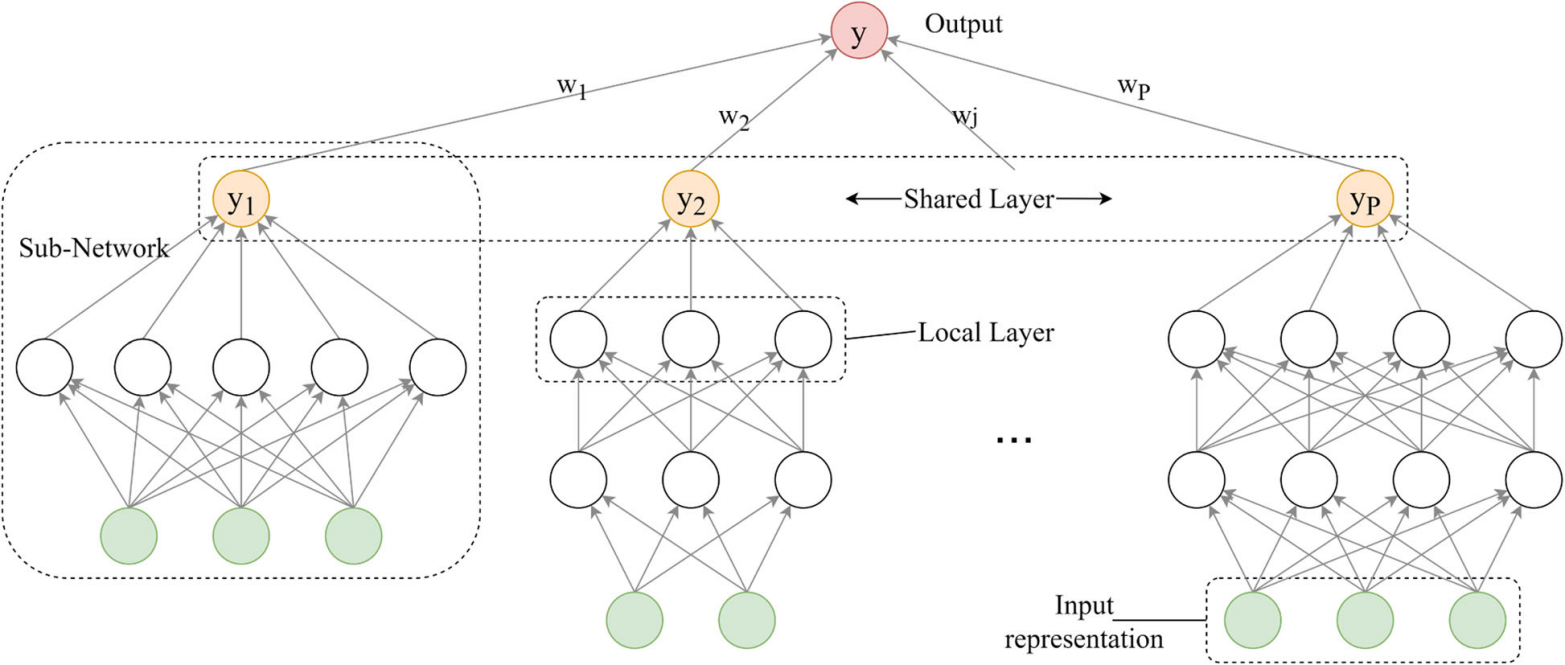
A Neural Network architecture to combine and integrate different sources and representations. Each base network is trained and validated on a single base representation. At the top of the network, a shared layer combines the output of the base networks. Green circles denote the input features. In the example, the network combines the output from *P* different neural networks, i.e. *P* different representations

Several strategies can be used for the combination of the methods above. The high-level combination mechanism described by Mioulet et al. [24] has been used in this work. The main idea is to define an ensemble consisting of shared and local layers. A single local network is created and validated for each base representation. Then, the output of these networks is combined by means of a shared layer. A scheme of the network is depicted in Fig. 2. Note that local networks can have different architectures, i.e. number of hidden layers and number of nodes.

The Keras [25] package with TensorFlow [26] as back-end has been used to implement the neural networks.

### CRAFT

The Colorado Richly Annotated Full Text (CRAFT) v2.0 corpus [12] contains a set of 67 full documents from the PubMed Central Open Access Subset. These documents have been manually annotated with respect to the following ontologies:

- Chemical Entities of Biological Interest (ChEBI) [27]: contains chemical names;
- Cell Ontology (CL) [28]: contains names of cell types;
- Gene Ontology (GO) [29]: the CRAFT corpus is annotated with two sub-categories, which are Cellular Components (GO_CC) and Biological Processes and Molecular Functions (GO_BPMF);
- National Center for Biotechnology Information (NCBI) Taxonomy [30]: includes names of species and taxonomic ranks;
- Protein Ontology (PR) : contains protein names;
- Sequence Ontology (SO) [31]: contains names of biological sequence, features, and attributes.

Globally, the CRAFT corpus contains more than 100,000 annotated concepts. The corpus has a further annotated entity type, i.e. NCBI Entrez Gene, which has not been considered in this work for two reasons. Firstly, the ontology contains several terms which overlap with frequent words, such as *“was”*, *“and”*. Moreover, the CRAFT distribution does not include a reference version for the terminological resource used to annotate Entrez Gene concepts. The same resource has been omitted in other works [3, 32]. The content of the CRAFT corpus is summarized in Table 1.

**Table 1:** CRAFT description

Entities	# Concepts
Chemical	7536
Cell types	5878
Gene Ontology	
Cellular component	21216
Biological process	8377
Organism	7453
Protein	15641
Sequence	21236

### Hybrid architecture

This work is based on the hybrid BNER architecture recently proposed by Basaldella et al. [3]. The system consists of a two-step pipeline which combines human knowledge with automatic learning algorithms.

The first phase of the system concerns the application of a dictionary look-up that scans the corpus and acts as an initial filter. This step is performed by means of the OGER annotator system [33]^2^. OGER combines several domain-specific dictionaries obtained from manually curated life-science knowledge bases through the Bio Term Hub (BTH) [34]^3^. These knowledge bases contain (among other things) all the names of entities of a number of predefined ontologies, and they are built by teams of linguistic and biomedical experts. The output of the dictionary look-up is a set of tokens from the corpus that match with an entry of the dictionary. These tokens define the set of *Candidates*, which are very likely to be entities of the selected ontology. Generally, the output of the dictionary look-up, i.e. the set of candidates, has a high recall but low precision. This means that there are few entities that are discarded by the dictionary, but also the set of candidates contains a lot of False Positives, i.e. tokens incorrectly selected.

The second phase of the system consists of a machine learning algorithm to filter further the set of candidates, aiming at increasing the precision while keeping high recall. Specifically, given a candidate entity retrieved by OGER, the algorithm predicts if the candidate is a biomedical entity or not (binary classification). We used the same strategy adopted by Basaldella et al. [3] to train this classifier. Given a set of training documents, we consider annotated entities as positive examples, whereas the false positive candidates selected by OGER constitute the negative examples.

Originally, the system contains two sets of features to describe a candidate entity in a vectorial space, consisting of grammar rules and affixes. The grammar rules have been defined by a group of experts, and they consist of presence/absence of capital letters, numbers, symbols and so on. These features are designed to emphasize the information useful to recognize biomedical names, and they are summarized in Table 2. Additionally, in the biomedical domain, affixes usually have a specific meaning, and they could have useful information to recognize relevant entities. For instance, the suffix *-ism* refers to particular conditions or diseases, such as *dwarfism*. Or the suffix *-ase* which is used to refer to an enzyme, such as *Acetylcholinesterase*.

**Table 2:** Grammar features. N: numerical; B: boolean

Feature	Type
num characters	N
is all uppercase	B
is all lowercase	B
contains Greeek letters	B
num dashes	N
num numbers	N
ends with a digit	B
contains capital letters	B
num lowercase characters	N
num uppercase characters	N
num spaces	N
num symbols	N

**Fig. 3:**
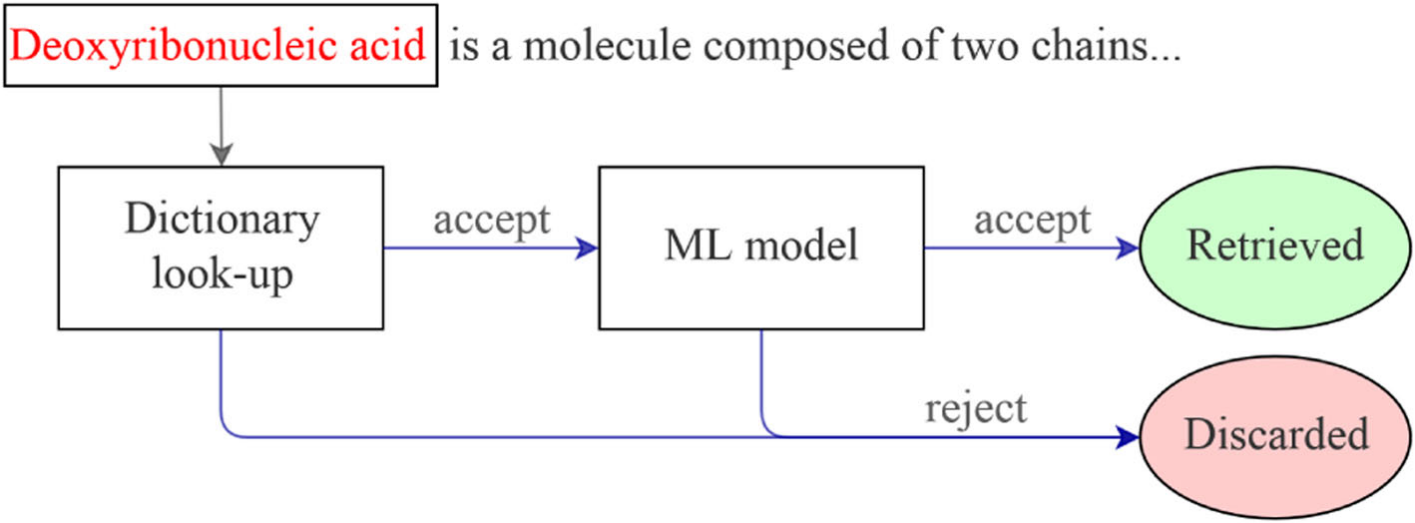
A depiction of the hybrid BNER system. A term is retrieved if it is selected by the dictionary look-up and it is accepted by the ML algorithm

A scheme of the system is depicted in Fig. 3.

### Learning from different sources

As introduced before, the main contribution of this work is to extend the hybrid architecture presented in [3], by learning the representation of entities as a combination of multiple representations. The combination mechanism is performed by the MKL algorithm or by the neural network shown in Fig. 2.

The proposed extension has two main advantages. Firstly, the solution that we propose relies on a more expressive feature set compared to [3]. Every base representation introduces a certain quantity of information useful to solve the task. Secondly, the principled combination of base representations provides a more flexible solution, where each feature set is re-weighted to better fit data according to a quality criterion. In the case of MKL, the criterion is related to a quality measure of the resulting representation, i.e. the margin in this case, whereas the neural networks are driven by the empirical measure which quantifies the errors, i.e. the loss function.

The combination allows to emphasize the most useful information, providing ad-hoc and adaptive solutions for each type of entities. Indeed, different entities may require different approaches. For instance, the recognition of a protein may require an observation of the affixes of the token, whereas diseases may be easily recognizable by observing the context. The recognition of different types of entities has different complexity, and they may require different and specialized representations. The definition of a single representation for the whole set of entity types may limit the expressiveness of the learning algorithm.

### Feature sets

The representations taken into account in this work are:

- Orthographic features: the representation defined in [3] and discussed previously. It consists of a set of manually defined features which describe the inner structure of a token, including the count of numbers, capital letters, dashes and so on. Table 2 shows the complete set of features.
- Affixes: 4 lists of two- and three-characters prefixes and suffixes have been extracted from 5 ontologies from the Bio Term Hub repository. These ontologies are Cellosaurus, Chemical compounds and diseases from the Comparative Toxicogenomics Database (CTD), Entrez Gene, Medical Subject Headings (MeSH), and Universal Protein Resource (Swiss-Prot). Then, a score is assigned to each possible affix, which represents the number of occurrences of such affix from a specific terminological resources. These features have been rescaled in [0,1]. Globally, 20 features have been computed (4 types of affixes times 5 resources).
- Word2Vec: is a shallow neural network widely used in NLP applications [10] to produce a distributed representation of input words (also known as work embedding). The network is designed as an encoder/decoder architecture, and it is trained with unsupervised strategies on large corpora. Given a word as input, the representation developed at the internal layer of the network describes the word and its meaning. Word2Vec is based on the concept that two words have a similar representation if they appear in the same contexts. In the remainder of the paper, we use the term Word2Vec to refer the representation that the netwoork produces rather than the network itself. These representations are general purpose, and the same learning procedure can be applied on several NLP tasks without prior domain knowledge and human effort on designing good representations. Two different Word2Vec representations have been included in this work^4^, which consists of models pre-trained on PubMed (domain-specific), and on Google news (general-purpose). The idea is to include two similar representations with different abstraction levels. We used the pre-trained word vectors without fine-tuning on the target data.
- Word-normalization features: the token is normalized, i.e., lower characters, upper characters, numbers, and symbols are transformed into 4 possible characters, that are ‘a’, ‘A’, ‘0’, and ‘-’. Then, for each of them, a set of features has been extracted, which are the total number of occurrences in the token, the maximum and minimum numbers of consecutive occurrences, and the total number of occurrences computed on the *compressed* token. The compressed token is a compacted version in which all the consecutive repetitions are removed (e.g.: the token ‘AAa0aaa’ becomes ‘Aa0a’).
- *p*-spectrum word-normalization: the normalized token is compacted by removing all repetitions. Then, features correspond to the presence or absence of all possible sub-strings with arity *p*. In this work, 5 different instances of this representation have been included, with arity from 1 up to 5.

These 10 representations contain different information to each other. Some of them consider the semantic information of a token in a possible context, focusing on the meaning of the word (word-level). Other representations instead consider the inner structure of the token and how it is composed (character-level). Moreover, some feature sets are more general than others, as is the case of Word2Vec pre-trained on news with respect to the version pre-trained on PubMed. Besides, the *p*-spectrum is the explicit representation of a well-known kernel for strings and sequences [35]. It counts the common substrings of a fixed length on two tokens. The combination of different *p*-spectrum representations simulates an embedded deep hierarchy of character-level features of increasing expressiveness. A categorization of these representations is described in Table 3.

**Table 3:** Representations description

Representation	Character level	Word level	Domain specific	Human designed	Automatically extracted
[3]	x		x	x	
affixes	x		x	x	
W2V - Pubmed		x	x		x
W2V - News		x			x
word normalization	x			x	
*p*-spectrum (x5)	x				x

### Model selection

Different strategies (and baselines) have been considered to integrate and to assess different feature sets. These strategies are:

- Single representation: the single feature set (i.e. the single base representation) is selected by using a canonical validation procedure. This baseline allows us to better understand the limits of considering a single representation at a time.
- Concatenation: the representation is defined as the vector concatenation of all base representations. In doing so, the resulting representation relies on a richer but static feature space, showing the advantages of multi-information.
- Combination: the proposed method. The representation is defined as a principled aggregation of all base representations.

**Fig. 4:**
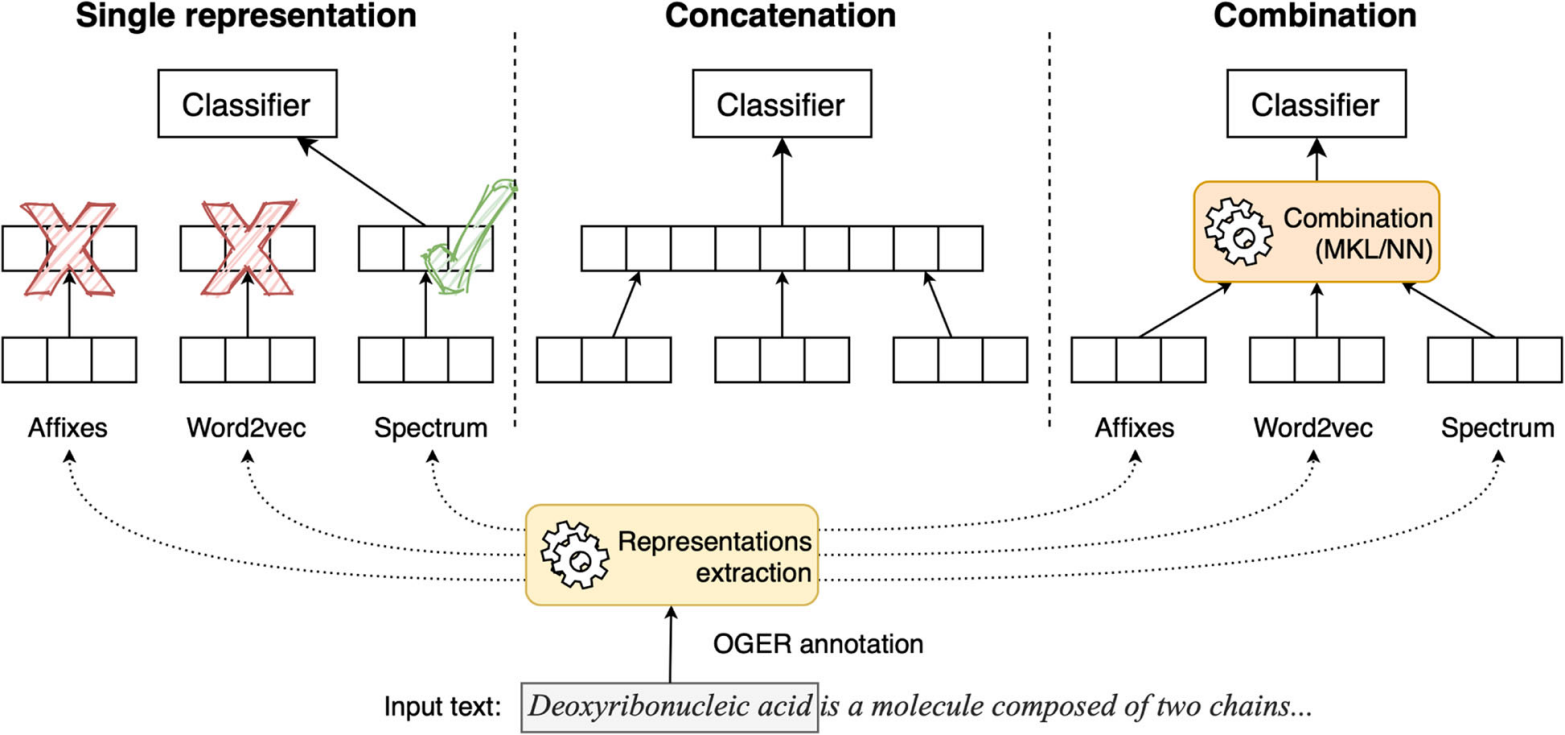
Depiction of the proposed system and other features aggregation schemes. The OGER annotator retrieves candidate entities from input texts (*Deoxyribonucleic acid* in the example). Then, different set of features associate to the candidate entity are computed (e.g. affixes and spectrum) or extracted (word2vec), producing multiple feature vectors. Consequently, the features aggregation schema defines the final representation as (i) a single base representation, (ii) the concatenation of base feature vectors, and (iii) the principled combination obtained through a MKL algorithm or a NN (shown in Fig. 2). The resulting representation is used with a classifier to select the final class (entity or not)

For each of the aforementioned mechanisms, both neural networks and SVM have been applied. These three schemes are described in Fig. 4.

The CRAFT corpus has been divided in 47 training and 20 test documents. The division is the same used in [3]. At the first training phase, the OGER system has been applied to the training documents, finding the set of candidate entities. The system has been used in its default configuration. Afterwards, the representations have been computed on the candidates. Then, a hold-out procedure has been applied to choose the hyper-parameters of the SVM and neural networks, by splitting the training candidates in training (80%) and validation (20%). The common hyper-parameters for Single representation, Concatenation, and Combination are:

- SVM: the regularization parameter $C \in \mathcal {C} = \{10^{i}, i= -5,\dots,5\}$;
- NN: the number of hidden layers in $\mathcal {D} = \{1\dots 4\}$, and the width of these layers, with values $h \in \mathcal {H} = \{0.5, 1, 1.5\}$ multiplied by the number of input features.

In the case of the single representation approach, the most suitable representation has been selected by performing a grid-search with the other hyper-parameters, with a consequent loss of efficiency. The asymptotic complexity of SVM and neural networks in this setting is $O(|\mathcal {C}| \times P)$ and $O(|\mathcal {D}| \times |\mathcal {H}| \times P)$ respectively, where *P* is the number of base representations.

The Concatenation method, instead, considers the concatenation of all the possible representations in a single one. In this way, the choice of the representation is not a hyper-parameter, and the models may exploit a more expressive feature set. The asymptotic complexity of SVM and NN becomes $O(|\mathcal {C}|)$ and $O(|\mathcal {D}| \times |\mathcal {H}|)$, respectively.

The Combination method is more complex. The architecture of the neural network is defined as an ensemble of small networks, each of them trained and validated with a single representation. Then, a shared layer is placed on top of these sub-networks. The schema of such architecture is depicted in Fig. 2. The training and the validation of such network is computationally expensive, and asymptotically comparable to the selection of the single representation. On the other hand, the EasyMKL algorithm has been used to combine these representations, providing the input for a hard-margin SVM. The *λ* hyper-parameter of the EasyMKL algorithm has been selected in validation, with values $ \lambda \in \Lambda = \{0.1, 0.2\dots 0.9, 1\}$. The computational complexity is *O*(|*Λ*|). In order to add non-linearity, in the case of MKL each base representation, with the exception of the spectrum, has been used both in their original (linear) form and polynomial, with degrees $2\dots 5$. We recall that the (homogeneous) polynomial kernel of degree *d* is computed as *k*(***x***,***z***)=〈***x***,***z***〉^*d*^.

## Results

When the classical validation procedure is used, the result of the classification depends only on the selected representation. In other words, there is a single representation which contributes to the classification. However, results show that the algorithms are able to exploit the richness of a global representation defined as a simple concatenation of base feature sets, where each of them contributes equally to the solution. Furthermore, dedicated combination mechanisms are able to adapt the representation on different tasks and domains, improving the accuracy of the classifiers, with some limited exceptions. Baselines and the combination methods have been compared in terms of *F*_1_ score, Precision and Recall. The achieved results are depicted in Table 4.

**Table 4:** *F*_1_ score (precision, recall) of the baselines and the proposed method, named Combination

	Single representation		Concatenation		Combination	
**Entity**	SVM	NN	SVM	NN	MKL	NN
chebi	**69.67**	**70.46**	**75.29**	**76.96**	**78.99**	**76.87**
	(88.84, 57.30)	(92.44,56.93)	(87.46, 66.08)	(83.91, 67.62)	(91.13, 69.70)	(90.1766.98)
cell	79.91	80.12	79.91	79.91	**80.16**	80.12
	(88.41, 72.91)	(88.92, 72.91)	(88.41, 72.91)	(88.41, 72.91)	(89.01, 72.91)	(88.92, 72.91)
go_cc	66.50	65.31	67.92	65.56	**68.92**	65.59
	(82.11, 55.87)	(81.92, 54.30)	(83.75, 57.12)	(84.31, 54.63)	(89.41, 56.06)	(82.81, 54.30)
go_bpmf	30.84	30.49	30.29	30.29	**36.20**	30.64
	(68.59, 19.89)	(69.72, 19.51)	(70.96, 19.26)	(70.32, 19.30)	(78.73, 23.50)	(71.34, 19.51)
organism	92.36	92.97	92.85	93.32	**94.99**	93.27
	(97.86, 87.45)	(99.24, 87.45)	(98.19, 88.07)	(99.35, 87.97)	(99.19, 91.13)	(99.19, 8.02)
protein	72.68	81.94	82.26	81.09	81.68	**84.38**
	(77.63, 68.33)	(81.41, 82.48)	(84.40, 80.23)	(85.53, 77.08)	(85.44, 78.23)	(88.73, 80.43)
sequence	72.26	72.61	71.77	72.47	**75.08**	72.51
	(89.58, 60.55)	(88.79, 61.42)	(88.67, 60.29)	(89.10, 61.07)	(93.11, 62.90)	(90.43, 60.52)

Best results are highlighted in bold characters

What is evident from the table is that the simple concatenation of base representations improves, on average, the performance of the system with respect to a single representation. In the case of SVM, the concatenation achieves better results on 4 entity types, which are chebi, go_cc, organism, and protein, whereas decreases the performance on go_bpmf and sequence. Neural networks instead have an unstable behaviour, and they improve the performance only on 3 entity types, which are chebi, go_cc, and organism. Chemical entities provide the largest improvement, that is +5.62 and +6.5% *F*_1_ for SVM and neural networks respectively. Concerning the combination mechanism, the MKL achieves always better results than the simple concatenation with the single exception of protein, where the *F*_1_ decreases from 82.26 to 81.68. Finally, MKL outperforms neural networks on 5 entity types.

In order to better explore the benefits of the proposed methodology, we analyzed the performance of the hybrid NER system by varying the individual base representations. Specifically, Table 5 shows, for a subset of entity types, the *F*_1_ score computed by the SVM when using a single base representation in rotation.

**Table 5:** *F*_1_ score computed when using a SVM with individual base representations for a subset of entity types

Representation	chebi	Protein	Sequence
Grammar	60.36	77.47	53.53
Affixes	56.06	74.03	53.53
w. norm	58.79	76.62	25.47
W2V-Pub.	69.67	72.68	**72.28**
W2V-News	**70.16**	**80.71**	72.26
Spectrum	54.47	73.15	53.53

Best results are highlighted in bold characters

As you can see, some individual representations, e.g. W2V, are better than other representations, e.g. spectrum. However, the principled combination improves the overall performance, meaning that “bad” representations still contain fruitful information.

Note that the validation procedure used to select the single best base representation (see Table 4) does not always provide the most suitable solution. For instance, the SVM applied to ChEBI entities encoded by W2V-News achieves 70.16 of *F*_1_ on the test set. However, the complete procedure achieves 69.67 as the validation performance computed by W2V-Pubmed is higher. The same holds for protein, where W2V-News achieves 80.71 of *F*_1_ on the test set whereas the complete validation produces a considerably lower result, i.e. 72.68. These results further emphasize the limits of a single-representation validation procedure in favour of a MKL solution.

### Weights evaluation

**Fig. 5:**
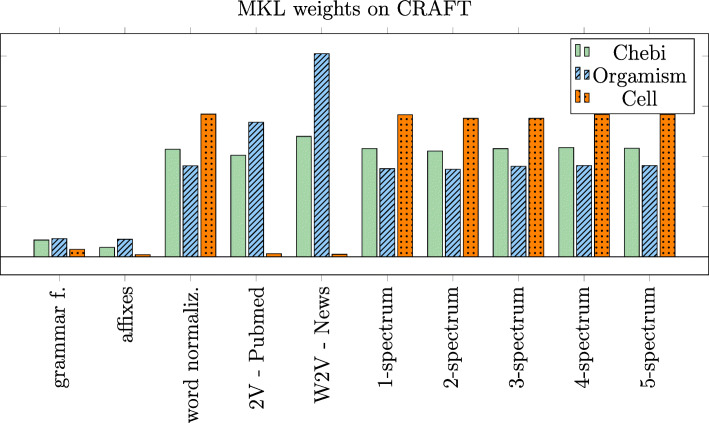
Contribution of base representations in the BioNER

The EasyMKL method learns and assigns a weight to each base representation, and each of them has its own contribution on the final results. The weight of the *r*-th representation is related to how much it contributes to the margin maximization, which corresponds to the learned weight *μ*_*r*_. A comparison of the learned weights on three different entity types from the CRAFT corpus is depicted in Fig. 5.

As shown in the figure, base representations cooperate differently in the combination depending on the task, and on the characteristics of the type of entities. Cell types are very specific terms, and the combination is focused on the n-chars representations, that jointly represent a deep character-level embedding and could represent better the inner structure of cells. Organisms, instead, exploit the word embeddings provided by the Word2Vec algorithm trained on both PubMed and news, whereas the contribution of the character-level representations is lower than for the other entity types.

A notable result is that the representations proposed in [3] received the lowest score on average. This result does not mean that these representations are wrong or badly built, but that they do not contribute as expected to the combined solution. This aspect is probably due to the fact that the same information is partially included into other representations. These results are an evidence of how representation learning techniques are fundamental in these systems. In any case, the constant trend of the weights assigned to the n-chars representations indicates that the solution requires character-level features with different arity, from 1 char up to 5. This result suggests that character-level deep representations are important on these tasks.

### Comparison against other systems

This work aims at understanding the benefits of feature combination procedures rather than comparing SVMs against neural networks, or our system against other architectures. However, Table 6 shows the performance in term of *F*_1_ of the proposed method and other recent architectures. In particular, the achieved results of three systems have been considered. The first is OGER, which has been used in this work only to perform a preliminary selection phase. The simple dictionary look-up achieves lower scores on average. Two notable results have been achieved by cell and organism entities. In the former case, OGER achieves results comparable to our system (79.91% vs. 80.16%), whereas in the latter case the machine learning filter doubles the performance (94.99% vs. 44.11%).

**Table 6:** Performance comparison (*F*_1_ score) of our method that uses the MKL as combination mechanism against other systems, including the recurrent architecture proposed by Crichton et al. [4], OGER, and fastText

Entity	Our sys.	Crichton et al.	OGER	fastText
chebi	**78.99**	74.83	53.73	75.24
cell	80.16	**86.89**	79.91	80.03
go_cc	**68.92**	63.08	54.63	67.99
go_bpmf	36.20	-	26.81	30.27
organism	94.99	**97.44**	44.11	92.64
protein	**84.38**	75.16	62.00	80.76
sequence	75.08	-	53.53	72.76

Best results are highlighted in bold characters

The second system (Crichton et al. [4]) which has been compared consists of a recurrent neural network. The idea is to compare the proposed solution against state-of-the-art architectures based on sequences.

As a further baseline, we consider our hybrid system that uses OGER as initial dictionary look-up and fastText [36] to extract a representation for the classification task. Notwithstanding fastText can be trivially considered as an additional base representation to augment our bag of representations, we consider the ensemble composed by OGER and fastText as an external system. In short, fastText is a popular algorithm to learn a distributed representation of words. Differently from Word2Vec, fastText represents a word as a bag of character n-grams. As a consequence, the representation produced by the model already includes both word- and character-level information, making fastText a relevant baseline to compare different combination mechanisms beyond MKL. In our experiments, we used an available fastText checkpoint pre-trained on Common Crawl^5^.

As you can see from Table 6, our system outperforms fastText on all entity types. This result shows that our MKL combination is significantly better than the fusion strategy used by fastText. Furthermore, we recall that fastText leverages extensive pre-training on large corpora, whereas our efficient architecture is only trained on target data, without any form of pre-training.

## Discussion

Despite the concepts analyzed in this work can be virtually applied to any NER/BNER system, we have considered the one proposed in [3] for several reasons. Firstly, the system has proven its effectiveness in the biomedical domain, achieving state-of-the-art results compared against other methods. Secondly, the layered architecture allows to easily inject in such system the multi-representation concepts, by tampering only the computation of the representation, without further specific adaptions. Then, the system/annotator is available on the web, and it can be used through specific API^6^ which can be easily modified to include multi-representations.

However, there are serious drawbacks of this approach. The first issue is the propagation of the errors. Entities that do not match with the dictionary look-up are not considered by the second layer, and the system will never retrieve them. Authors in [3] tried to overcome this problem by considering the dictionary look-up as a further feature instead of a hard filter, without significant improvements.

The second problem concerns the need of the dictionaries. On the one hand, dictionaries are strictly domain-dependent, and their update requires a lot of human effort. Moreover, there is a further problem with the versioning of the dictionaries. The first phase of the architecture fails when the version of the dictionary is not aligned with the corpus. In other words, the look-up does not work well if it is applied to an old annotated corpus with a recent dictionary. On the other hand, the dictionaries are valuable resources which contain useful information, essential in the biomedical domain. The last limitation of this system is that it does not take into account the context. However, this point could be solved by introducing a further context-dependent representation.

However, aware of these limits, this paper is focused on a different problem, that is the choice of the representation used to model entities and candidates.

### Related work

Several methods have been developed in the past decades for NER.

Early NER systems were based on hand-crafted rules, linguistic and orthographic features, and ontologies. On the one hand, these methods do not require annotated corpora or expensive computational resources. On the other hand, they rely on linguistic expertise for designing effective rules, and human effort on updating the ontologies, a critical aspect especially in the biomedical scenario, where novel terms emerge frequently.

In the last decade, these systems have been replaced by machine learning methods, mainly based on SVM [37, 38], CRF [6, 7, 39], and, more recently, by deep Neural Networks [4, 40, 41], showing a significant increase of accuracy.

Recently, state-of-the-art methods deal with the NER task as a word-sequence labeling problem. These systems rely on a convolutional or bidirectional Long-Short Term Memory (LSTM) layer applied to sentences [42], sometimes with an additional CRF layer [43]. Input sequences are usually sentences, where atomic words are represented by word-embeddings, such as the well-known Word2Vec [10]. The main idea behind these methods is that word-level features are important, but they are not able to solve problems related to the disambiguation without considering the context, caught by the whole sentence.

Lately, character-level features have been included in these architectures, showing their benefits on several corpora. An empirical comparison between a set of hand-crafted features and the Word2Vec embedding trained on PubMed has been proposed in [44]. See [45] for a recent and exhaustive survey on deep and neural network based NER methods.

Finally, recently Transformer models pre-trained on Biomedical corpora [46] have been applied to the BioNER task, with remarkable results compared against previous methods. For instance, Xin Yu et al. [47] showed that the Transformer improves the simple Bidirectional LSTM with CRF by 3 points of *F*_1_ on electronic medical records. Similarly, Symeonidou et al. [48] showed an improvement of 10.5 points of *F*_1_ in BNER for adverse drug reaction recognition.

However, despite the empirical effectiveness and the capability of recurrent architectures and Transformers, hybrid systems based on both machine learning techniques and dictionary look-up are receiving much attention in the literature. Some noteworthy examples of such hybrid systems are [3, 49–52]. Other systems try to combine rule-based approaches to machine learning methods, as is the case of [53, 54].

Hybrid systems have some advantages and strengths. Firstly, the training of such models require less annotated data than deep neural networks. Thus, these systems can be easily applied to low-resources scenarios. Second, the simple retrieval of an entity is usually not sufficient, and there is the need of linking the retrieved entity to the concept that it represents. This step is simple in the case of dictionary look-up methods. Finally, especially in the biomedical domain, entities are particular terms which are easily codified in dictionaries and ontologies.

## Conclusions

Several architectures exist in the literature to extract relevant entities from the biomedical literature. However, one of the main problems of these systems is the choice of the data representation.

In this paper a thorough analysis of the existing representations has been conducted, showing three different methodologies to consider and to aggregate different sets of features. These methods are the selection of the single representation through a model selection step, the concatenation of representations, and the principled combination. These approaches have been analyzed by using both neural networks and Support Vector Machines. Several types of representations have been used. Some of these are based on a strong prior knowledge of human experts, others consist of neural embeddings or general purpose word vectors. Moreover, a two layered Entity Recognition system has been used as a proof-of-concept of the proposed methodology. This architecture exploits the human knowledge encoded in dictionaries to improve the automatic classification.

The results show that the cooperation between these representations improves the accuracy of correct classification. The concatenation, which corresponds to the average of base kernels for the SVM, achieves better results than the simple selected representation. The concatenation is known to be a hard baseline, which takes advantage when the base representations achieve good results singularly. The combination mechanisms achieve on average better results than the concatenation and the single representations. Finally, we compared our method against a recent architecture based on recurrent neural networks, showing comparable results.

In the future, we plan to apply our proposed methodology to different BNER architectures, aiming at overcoming the limits of the dictionary look-up. Moreover, other feature sets will be included which contain document-level and sentence-level information. The former provides information from the main topic of the document, the latter implies contextual information of the entity.

## Data Availability

The corpus CRAFT [12] used in this paper is freely available, and it can be found at http://bionlp-corpora.sourceforge.net/CRAFT/.

## Abbreviations

NER: Named entity recognition
BNER: Biomedical named entity recognition
CAT: Computed aided tomography
HIV: Human immunodeficiency virus
NLP: Natural language processing
SVM: Support vector machine
CRF: Conditional random fields
RKHS: Reproducing kernel Hilbert space
MKL: Multiple kernel learning
NN: Neural network
CRAFT: Colorado richly annotated full text
BTH: Bio term hub
LSTM: Long short term memory

## Footnotes

1 https://github.com/IvanoLauriola/MKLpy

2 http://www.ontogene.org/resources/oger

3 http://www.ontogene.org/resources/termdb

4 Available on https://code.google.com/archive/p/word2vec/

5 https://fasttext.cc/docs/en/english-vectors.html

6 inserire url
